# Plasma HIV-1 tropism and risk of short-term clinical progression to AIDS or death

**DOI:** 10.7448/IAS.17.4.19685

**Published:** 2014-11-02

**Authors:** Maria Casadellà Fontdevila, Alessandro Cozzi-Lepri, Andrew Phillips, Marc Noguera Julian, Marcus Bickel, Dalibor Sedlacek, Gitte Kronborg, Adriano Lazzarin, Kai Zilmer, Bonaventura Clotet, Jens D Lundgren, Roger Paredes

**Affiliations:** 1 IrsiCaixa AIDS Research Institute, Barcelona, Spain; 2 Royal Free Hospital, London, UK; 3 Goethe University, Frankfurt, Germany; 4 Charles University Hospital, Plzen, Czech Republic; 5 Hvidovre, Copenhagen, Denmark; 6 Ospedale San Raffaele, Milano, Italy; 7 West-Tallinn Central Hospital, Tallinn, Estonia; 8 Panum Institute, Copenhagen HIV Programme, Copenhagen, Denmark

## Abstract

**Introduction:**

It is uncertain if plasma HIV-1 tropism is an independent predictor of short-term risk of clinical progression / death, in addition to the CD4 count and HIV RNA level. We conducted a nested case-control study within EuroSIDA to assess this question amongst people with current HIV RNA level >1000 copies/mL, including both people on ART and those ART naïve.

**Methods:**

People with an AIDS diagnosis or who died from any causes for whom there was a stored plasma sample with HIV-1 RNA (VL)≥1,000 copies/mL available in the time window of 3–12 months prior to the event were identified. At least one control was selected for each case matched for age, VL and HCV status at the time of sampling. Controls were event-free after a matched duration of time from the date of sampling. Plasma HIV tropism was estimated using 454 and population sequencing (PS). Non-R5 HIV was defined as: (a) ≥2% of sequences with a Geno2Pheno (G2P) FPR≤3.75% by 454, and (b) a G2P FPR≤10% by PS. We also compared CD4 slopes over the 12 months following the date of sampling using a linear mixed model with random intercept according to HIV tropism and ART status.

**Results:**

The study included 266 subjects, 100 cases and 166 controls, with sample taken on average in 2006; 23% and 24% had non-R5 HIV by 454 and PS respectively. There were 19% women, 25% MSM, 92% Caucasians, 22% HCV+. At the time of sampling, 26% were ART-naïve, 25% had started but were off ART and 49% were receiving ART. The median age, CD4 and viral load was 41 years, 350 cells/mm^3^ and 4.81 log c/mL, respectively. Baseline characteristics were well balanced by tropism. Factors independently associated with clinical progression or death were female gender (OR=2.12; 95% CI=1.04, 4.36; p=0.038), CD4+ count (OR=0.90 per 100 cells/mm^3^ higher; 95% CI 0.80, 1.00; p=0.058), being on ART (OR=2.72; 95% CI 1.15, 6.41; p=0.022) and calendar year of sample (OR=0.84 per more recent year; 95% CI=0.77, 0.91; p<0.001). Baseline plasma tropism was not an independent risk factor for clinical progression or death by either 454 or PS. No significant interaction was observed between tropism and ART status. There were no significant differences in the CD4+ slope within or between tropism groups.

**Conclusions:**

Plasma HIV-1 tropism does not appear to add to the ability of CD4 count and viral load to predict the short term risk of AIDS and death outcomes, even with 454 sequencing.

